# Supplementary material for: An Open-Source Web-Based Viewer for Disseminating Image-Derived Models
Source: ArXiv. 2026 Oct 19:arXiv:2610.19237v2. Preprint. [Version 2] (PMC13738915)
Supplement: Supplementary file 1 [file NIHPP2610.19237v2-supplement-1.pdf]

## Online Resource Captions

**Online Resource 1** Video overview of representative clinical cases in the web-based Viewer, including procedural planning models and time-resolved CT, MRI, and 4D flow visualizations. 4D=4-dimensional; CT=computed tomography; MRI=magnetic resonance imaging

**Online Resource 2** Video demonstration of the web-based Viewer on a tablet, showing touch and stylus interaction with representative procedural planning models and time-resolved imaging visualizations.
